# Colovaginoplasty in a Case of Mayer-Rokitansky-Kuster-Hauser Syndrome

**Published:** 2014-04-01

**Authors:** Muhammad Saleem, Muhammad Zafar Iqbal, Mazher Rafee Jam, Mushtaq Ahmad, Bilal Mirza

**Affiliations:** Department of Pediatric Surgery, The Children’s Hospital and the Institute of Child Health Lahore, Pakistan; Department of Pediatric Surgery, Sheikh Zayed Hospital Rahim Yar Khan, Pakistan; Department of Pediatric Surgery, Sheikh Zayed Hospital Rahim Yar Khan, Pakistan; Department of Pediatric Surgery, Sheikh Zayed Hospital Rahim Yar Khan, Pakistan; Department of Pediatric Surgery, The Children’s Hospital and the Institute of Child Health Lahore, Pakistan

**Keywords:** Mayer-Rokitansky-Kuster-Hauser Syndrome, Vaginal agenesis, Colovaginoplasty

## Abstract

Mayer-Rokitansky-Kuster-Hauser Syndrome (MRKHS) is characterized by various abnormalities of paramesonephric duct structures; vaginal aplasia being the commonest anomaly in the spectrum. We report a 17-year-old girl; a case of MRKHS with vaginal agenesis. The cervix was present but atretic; uterus, fallopian tubes and ovaries were normal. There were no associated renal or skeletal defects. Colovaginoplasty was done to bridge the gap between uterus and introitus. Postoperatively, small part of colovaginoplasty flap became necrotic posteriorly, which was ultimately managed by insetting of labial flap.

## INTRODUCTION

Vaginal agenesis is a rare condition with an incidence of 1 in 4000 to 1 in 10000 live births; the most common etiology being mullerian agenesis.[1-3] About 15% of the patients with amenorrhea presenting to gynecological outpatient department have MRKHS. Uterus may be normally developed or it may be rudimentary bi-cornuate without lumen or it may totally be absent. Vaginal agenesis may also occur as an isolated abnormality.[4] Treatment of vaginal agenesis, in case uterus is present, is by substituting with isolated bowel segment that has shown excellent results.[5] Various problems of colovaginoplasty have been reported.1-6] Herein, we report a case of MRKH syndrome.

## CASE REPORT

A 17-year-old girl presented with crampy lower abdominal pain for last five years and primary amenorrhea. The pain was cyclical in nature usually occurred after every month, gradual in onset, progressively severe in intensity, remained for 4 to 5 days and often relieved with medicine. Family history was insignificant. She was otherwise generally healthy with no significant past medical history. Her height was 142 cm, weight 45 kg and blood pressure 120/70 mmHg. Her breast and pubic hair were at Tanner stage 4. Examination revealed an ill defined mass palpable in the lower abdomen. Genital examination disclosed normal appearing genitalia with a vaginal dimple only 1 cm deep. Ultrasound abdomen revealed a normal sized anteverted uterus with normal appearing endometrium (10 mm thick); cervix appeared hypoplastic however vagina was not outlined; both ovaries were normal. A tubular structure sized 8.1 cm x 3.6 cm with internal echoes and debris representing hematosalpinx was also noted. CT scan showed absence of vagina, hypoplastic cervix, and left hematosalpinx (Fig. 1).

**Figure:**
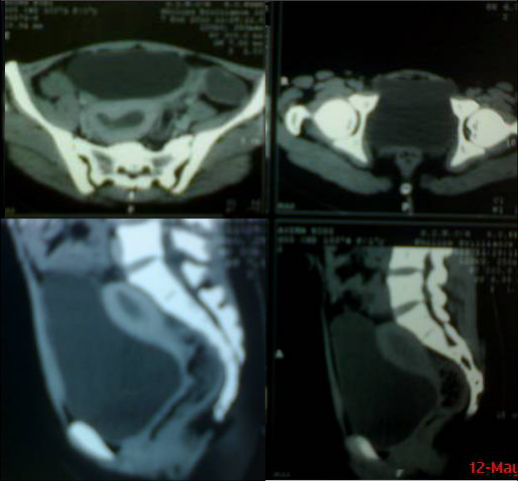
Figure 1:CT scan showing dilated uterus in upper right film. Lower vertical sections showing bladder with adjacent rectum inferiorly and uterus superiorly. No vaginal tissue below uterus is apparent.

Bowel vaginoplasty to bridge the gap between the uterus and introitus was planned. At operation, findings were a large bulky uterus, left hematosalpinx with occluded fimbriae. There was endometriosis of left fallopian tube and ovary. Right fallopian tube although edematous looked normal and so was right ovary (Fig. 2). Cervix was hypoplastic ending blindly having no vagina. Hystrotomy at the fundus was performed and patency of cervix checked. Cervix at the lower most patent part was then opened. The sigmoid patch was harvested, though sigmoid mesentery was short, as a vaginal conduit. A space was created between the bladder and rectum from above and from below at normal site of vagina in the vestibule. The conduit was brought down behind the left tube isoperistaltic fashion. It was anastomosed to uterus above and introitus below (Fig. 3, 4A). At Examination under anesthesia (EUA) on 3rd postoperative day, the lower posterior part of neo-vagina partly necrosed which was initially debrided and then replaced by labial flap after 2 weeks of initial surgery (Fig. 4B). EUA afterwards confirmed the viability and patency of neo-vagina. Patient is on regular vaginal dilatations. Passage of normal menstruation further confirmed the patency.

**Figure:**
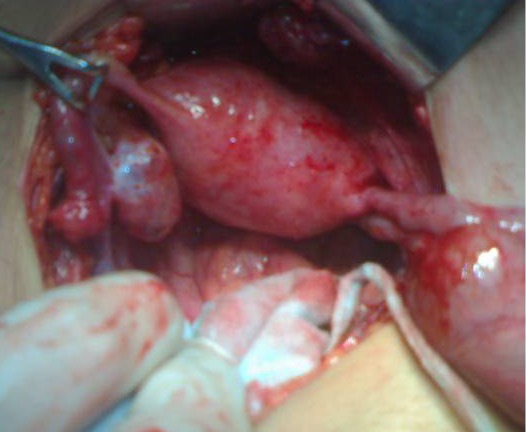
Figure 2:Large bulky uterus, left hematosalpinx with occluded fimbriae are noted.

**Figure:**
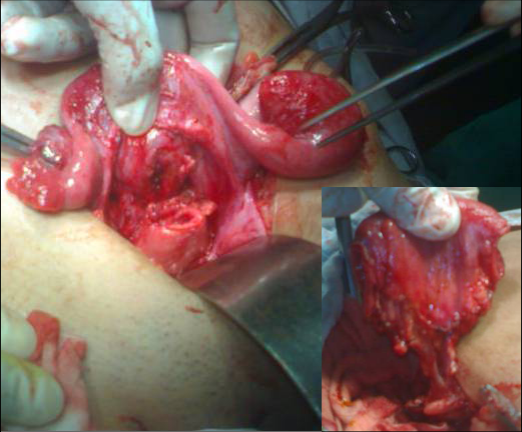
Figure 3:Harvested sigmoid colon (Inset), for colo-cervical anastomosis.

**Figure:**
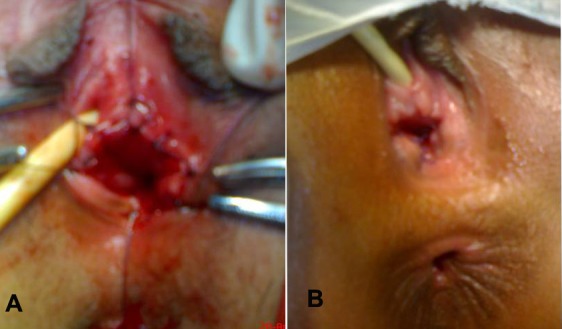
Figure 4:A, Completed procedure. B, At 2 weeks follow up after insetting a labial flap.

## DISCUSSION

MRKHS is described for the first time by Mayer in 1829; Rokitansky in 1838 and Kuster in 1910 further elaborated the presence of rudimentary uterus along with absence of vagina: in 1961, Hauser et al added association of renal and skeletal anomalies. These patients usually present at time of puberty with primary amenorrhea with or without cyclical cramping abdominal pain. These patients are 46 XX females; rarely may be associated with Turner syndrome or mixed gonadal dysgenesis. Depending upon the presence or absence of other malformations, the syndrome is divided into; typical (type 1- isolated form of congenital agenesis of the vagina and uterus) and atypical (type 2- having other associated anomalies of genital, renal, ear and skeletal).[1-5] Our patient was of typical MRKHS variety.

The goal of therapy is to provide adequate sexual function, provide a patent route to pass menstruation and conceive naturally in case of adequately functioning uterus, and deal with the psychological issues. A large number of procedures both operative and non operative have been advocated for creation of neo-vagina.[7] Surgical options are tailored as to presence or absence, and state of uterus and of segment of vagina. In index case sigmoid colon was used for reconstruction of neo-vagina because of its added benefits despite the fact that its mesentery was short in our case. This probably led to necrosis of posterior part of neo-vagina. The procedure worked well for the patient as her symptoms are abated.

## Footnotes

**Source of Support:** Nil

**Conflict of Interest:** None declared

